# The Communication, Awareness, Relationships and Empowerment (C.A.R.E.) Model: An Effective Tool for Engaging Urban Communities in Community-Based Participatory Research

**DOI:** 10.3390/ijerph14111422

**Published:** 2017-11-21

**Authors:** Joniqua Ceasar, Marlene H. Peters-Lawrence, Valerie Mitchell, Tiffany M. Powell-Wiley

**Affiliations:** 1 Cardiovascular Branch, National Heart, Lung and Blood Institute, National Institutes of Health, Bethesda, MD 20892, USA; joniqua.ceasar@nih.gov (J.C.); valerie.mitchell@nih.gov (V.M.); 2 Blood Epidemiology and Clinical Therapeutics Branch, Division of Blood Diseases and Resources, National Heart, Lung and Blood Institute, National Institutes of Health, Bethesda, MD 20892, USA; mpeters@nhlbi.nih.gov

**Keywords:** community-based participatory research, mobile health technology, recruitment, Black/African American, underrepresented populations, health disparities, public health and health promotion

## Abstract

Little is known about recruitment methods for racial/ethnic minority populations from resource-limited areas for community-based health and needs assessments, particularly assessments that incorporate mobile health (mHealth) technology for characterizing physical activity and dietary intake. We examined whether the Communication, Awareness, Relationships and Empowerment (C.A.R.E.) model could reduce challenges recruiting and retaining participants from faith-based organizations in predominantly African American Washington, D.C. communities for a community-based assessment. Employing C.A.R.E. model elements, our diverse research team developed partnerships with churches, health organizations, academic institutions and governmental agencies. Through these partnerships, we cultivated a visible presence at community events, provided cardiovascular health education and remained accessible throughout the research process. Additionally, these relationships led to the creation of a community advisory board (CAB), which influenced the study’s design, implementation, and dissemination. Over thirteen months, 159 individuals were recruited for the study, 99 completed the initial assessment, and 81 used mHealth technology to self-monitor physical activity over 30 days. The culturally and historically sensitive C.A.R.E. model strategically engaged CAB members and study participants. It was essential for success in recruitment and retention of an at-risk, African American population and may be an effective model for researchers hoping to engage racial/ethnic minority populations living in urban communities.

## 1. Introduction

The obesity epidemic contributes directly to the cardiovascular health of the U.S. population. Obesity promotes an increased prevalence of diabetes, hypertension, and dyslipidemia, and also directly leads to excess cardiovascular morbidity and mortality of the U.S. population [1,2,3]. Additionally, obesity has been thought to be a contributor to the recent reductions in the life expectancy in the United States [4].

The American Heart Association (AHA) and governmental health agencies have established goals for improving the cardiovascular health of the U.S. population by the year 2020, based on cardiovascular health factors. These goals include improvements in body mass index (BMI), physical activity (PA), dietary intake, total cholesterol, blood pressure, fasting plasma glucose, and cigarette smoking cessation [5,6]. Of the modifiable cardiovascular health factors, excess weight is associated with inadequate PA and poor dietary habits that promote worsening of blood pressure, glucose, and lipids [7,8]. Thus, interventions to decrease excess weight through increased PA and greater adherence to dietary guidelines would likely improve all cardiovascular health factors excluding cigarette smoking. 

Community-based interventions may address disparities in cardiovascular health across racial/ethnic groups, particularly among those with limited access to care in clinical settings [9]. Community-based participatory research (CBPR) principles are frequently used to engage at-risk communities in intervention development and implementation, with a community-based health and needs assessment being a first step in identifying targets for intervention [10]. However, very little is known about effective methods for recruitment and retention of at-risk, racial and ethnic minority populations, who have traditionally been difficult to recruit into clinical research studies, for community-based health and needs assessments. Additionally, very little is known about best practices for engaging individuals in community-based health and needs assessments that incorporate mobile health (mHealth) technology.

The Washington, D.C. Cardiovascular Health and Needs Assessment was designed by a team of investigators from various institutes at the National Institutes of Health (National Heart, Lung and Blood Institute (NHLBI), National Human Genome Research Institute, Clinical Center, and National Institute of Diabetes and Digestive and Kidney Disease), Howard University in Washington, D.C., church leaders from Washington, D.C., and members of the D.C. Cardiovascular Health and Obesity Collaborative (DC CHOC) community advisory board. The study was designed in 2012, Institutional Review Board approved in 2013 and led by principal investigator (PI) Dr. Tiffany Powell-Wiley to: (1) evaluate cardiovascular health factors (i.e., PA, dietary intake, cigarette smoking, BMI, total cholesterol, blood glucose, and blood pressure); (2) to assess usage of mobile health (mHealth) technology for objectively measuring PA and dietary intake and usage of web-based technology for monitoring cardiovascular health factors; and (3) evaluate environmental, cultural, and psychosocial factors that might help or hinder health behavior change. This health and needs assessment was funded by the Division of Intramural Research at the NHLBI of the National Institutes of Health and implemented as a first step in the development of a community-based health behavior intervention for populations from predominantly African American churches in resource-limited communities of Washington, D.C. Wards 5, 7, and 8, areas where neighborhood-level socioeconomic status is lowest and resources for healthy behaviors are most limited. 

**Figure 1:**
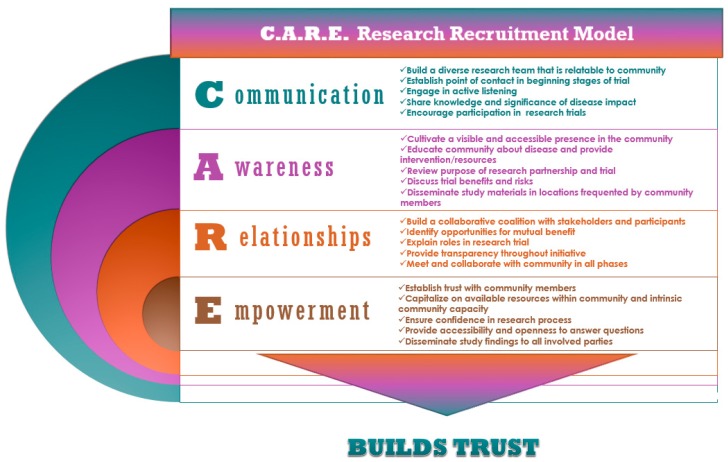
The C.A.R.E. (Communication, Awareness, Relationships and Empowerment) Research Recruitment Model. The culturally and historically sensitive C.A.R.E. Research Recruitment Model may be an effective means of recruiting and retaining predominantly Black/African American populations living in urban communities.

The Communication, Awareness, Relationships, and Empowerment (C.A.R.E.) Research Recruitment model was designed by a member of our research team to systematically address barriers to recruitment and retention of study participants (Figure 1). Each element of the C.A.R.E. model was used in building collaborations with community partners in this CBPR project. The C.A.R.E. strategies were also implemented at various stages of this study—from recruitment through implementation to dissemination. We hypothesized that the use of the C.A.R.E. model would allow us to (1) meet our target of recruiting and enrolling 100 eligible participants for the study; and (2) retain over 50% of the participants in the use of mHealth technology throughout the study. 

## 2. Materials and Methods 

### 2.1. Using the C.A.R.E. Model

Each part of the C.A.R.E. model involved specific strategies that were implemented throughout various stages of the study. 

### 2.2. Communication

The study’s infrastructure was built with the primary goals of ensuring that the team would successfully recruit and retain participants, and be composed of team members who would be engaged with and received well by the community. Team members were chosen for their specific areas of professional expertise, their interest and knowledge of the culture of African American churches, and their experience working with African Americans living in high-risk communities. Furthermore, to implement a study in this community, the assembled research team was racially/ethnically and culturally diverse.

In addition to the PI, the research team included academic researchers from Howard University, National Institutes of Health (NIH) post baccalaureate research fellows, a research nurse, and an outreach coordinator (OC). The team was further enhanced with volunteers: NIH nurses, a medical technologist, and Howard University graduate students who supported the data collection events. The OC promoted the vision and mission of the study in the community with support and participation of the entire research team and, most importantly, from church liaisons. Each participating church designated a liaison as the main contact person between the church and the research team. The church liaisons became study ambassadors and were key voices in the church community by communicating our mission, supporting our recruitment and retention efforts, and providing assistance for mHealth technology usage. 

The research team took the lead in forming partnerships that would facilitate active listening to ensure that our goals aligned with the community’s goals and in establishing points of contact for the community early on so that interested individuals would know exactly who to contact with questions about the study. The importance of active listening was evinced early when the Community Advisory Board (described in more detail under Relationships) suggested having initial focus groups as means for the community to participate in the research design. Eight individuals from the target population were recruited to take the survey and test the device to ensure that the study instruments (survey and PA tracker) conveyed our intended meanings. The feedback received from the focus group participants provided feedback to the study group that identified possible barriers in completing the survey, such as unclear language in the survey questions [11].

Our team also made it a priority to communicate and share knowledge and significance of cardiovascular disease impact by attending community events where potential participants could be recruited for the upcoming study. 

### 2.3. Awareness

Our team intentionally cultivated a visible and accessible presence in the Washington, D.C. health community of Wards 5, 7 and 8, by attending and participating in networking events with local government health agencies, health-focused community organizations and churches. As we became known and visible in the community, we were invited to attend health advocacy meetings in each of the targeted wards, to participate in health fairs and community events, and to provide support for health-related initiatives. We took advantage of these opportunities in the community to provide the community members with cardiovascular disease awareness information using culturally tailored flyers and posters and to recruit potential study participants (Supplemental Figure S1).

To ensure that our participation in community events was beneficial to individuals in the community, we provided free personalized cardiovascular disease risk assessments. These assessments were performed using a mobile application (Zuum, St. Louis, MO, USA, Supplemental Figure S2). Using this application, individuals received a quick description of their personal cardiovascular disease risk and customized tips on cardiovascular disease prevention. 

Community outreach activities allowed us to gain access to various segments of the targeted community, who we otherwise may not have been able to reach, and network with community-based advocacy organizations and local government officials. From these encounters, we recruited members for our community advisory board (CAB) and study participants. When interacting with potential study participants, we were able to discuss our goals, highlight benefits to participants, and share our contact information with those interested. Attendance at advocacy meetings and local government forums allowed us to remain informed about health-related activities in the community and provided a venue to disseminate information about the purpose of this research partnership and the community-based health assessment and the assessment’s risks and benefits.

### 2.4. Relationships

#### 2.4.1. Church Partnerships

Faith-based organizations were selected to be our hubs in the community because of their historical success as effective sites for community-based interventions targeting lifestyle changes within the African American community [12]. Several churches located in D.C. Wards 5, 7 and 8 were invited to participate in the study, with six churches accepting the invitation. The only criterion for a church’s participation was internet accessibility at the church facility. The initial contact with the churches was with the church Pastor and senior leaders to obtain approval to work with their congregation at their facility. The reasons that churches gave when they declined the invitation for participation were either conflicting church events or previous health initiative commitments. The most cited reason pastors gave for wanting their church to participate in the study was because they recognized the problem of poor cardiovascular health in their community and they were interested in helping their congregation make lifestyle/behavior changes related to PA and dietary intake. All of the church partners had an active health ministry or were interested in developing a health ministry. To help the congregations get to know the NIH research team better, we gave an informational presentation during church services, placed announcements in church bulletins, and distributed brochures/flyers about cardiovascular disease and our research. 

Church/Research Team relationships were maintained with our frequent visibility at the churches, supporting their health-related activities before, during and after the study assessment. Church ambassadors supported participant recruitment and encouraged retention but also provided the capacity for us to reach greater numbers of people in the community, connecting us to networking opportunities with other organizations.

#### 2.4.2. Community Partnerships—Community Advisory Board

Community engagement has been an on-going, mutually beneficial relationship that we have maintained through frequent, personal face-to-face contacts, such as attendance at church events and community health advocacy meetings. To overcome the initial barrier of being perceived as outsiders, we looked to faith-based organizations who wanted to improve health in their congregations, academic institutions, and government agencies with a health focus to partner with us as we developed a Community Advisory Board (CAB), which was called the DC Cardiovascular Health and Obesity Collaborative (DC CHOC).

### 2.5. Empowerment

As a core element of this study, DC CHOC participated in the planning and implementation of the health and needs assessment, interpretation and dissemination of study findings, and is participating in the design of a subsequent community-based health behavior intervention developed based on the health and needs assessment findings. Engaging the C.A.R.E. model, CAB members are recruited on an ongoing basis through networking at community events, recommendations from the community, other CAB members, study participants and NIH staff. Our CAB includes community members, representatives from the Washington, D.C. faith-based community, local and federal government agencies, academia and health advocacy organizations, all of whom are interested in addressing obesity and cardiovascular health in Washington, D.C. at-risk communities. 

Quarterly CAB meetings are held at different locations in the community, including churches, health care facilities, and academic institutions. The agenda includes briefings on the study development, dissemination of the projects’ findings, and announcements of related health advocacy events held in the community. CAB members share their perceptions, opinions, beliefs, and attitudes about the development and outcomes of the study, which helps our research team engage in active listening and identify opportunities for the mutual benefit of all entities involved. 

The empowerment portion of the C.A.R.E. model was implemented to be sure that participants’ involvement in the study was beneficial. Participants received a “Know Your Numbers” record card documenting their personal cardiovascular health measurement data obtained at the assessment event and with space for tracking additional measures of blood pressure, labs and anthropometric measurements. Participants also received a private consultation with the PI, a cardiologist, which included follow-up referrals when necessary. Finally, they received nutritional informational sessions that were delivered during wait times between stations by a nutritionist with culturally-tailored handouts that encouraged healthy lifestyle choices.

#### 2.5.1. Study Design and Implementation in Collaboration with CAB

Based on recommendations from the CAB, we implemented a two-week pilot to assess the functionality of the study design. The final design was implemented at six data collection events at four Washington, D.C. churches located in Wards 5, 7 and 8 and held from September 2014 through February 2015. Participants visited six stations at the events: (1) final registration and informed consent; (2) blood pressure measurement, blood sample collection, and anthropometric measures; (3) survey instrument completion; (4) mHealth device training; (5) cardiovascular risk assessment with the PI; and (6) debriefing. More information about the study design and findings have been documented elsewhere [13,14]. 

Briefly, the data collection events measured blood pressure and BMI. Blood samples were collected to analyze fasting blood glucose and plasma lipids. Cardiovascular health behaviors including alcohol and tobacco use, fruit and vegetable consumption, PA and sedentary behavior were captured using a culturally-tailored survey instrument. 

#### 2.5.2. Mobile Health Technology

Physical activity (PA)-monitoring wristbands were given to participants to wear for one month during the study. During the health and needs assessment, they received face-to-face training on the use of the wristband device and obtained a written instruction manual. Additionally, participants were provided with a device training video they could access from a home or church-based computer. The wrist-worn PA monitor collected accelerometer-based data on their amount of PA and intensity (e.g., steps taken, calories burned, distance travelled and minutes of vigorous activity). 

A hub was installed in an easily-accessible location at each church. Participants uploaded their PA data from their wristband devices to the church-based centralized hub once a week during the one-month study to synchronize their wristbands with the hub. After successfully synchronizing their wristbands with the hub, participants had access to their recorded accelerometer-based PA data in addition to all self-logged data on the study’s website.

Participants were provided a secure account (using a de-identified username and password) on a website associated with the PA-monitoring wristband. Training was given on general website use and how to log self-reported PA, weight, dietary intake, heart rate, blood pressure and blood glucose levels. A computer was available in the church for participants, if needed, to access their PA data during training and over the month follow-up period. The research team obtained wristband usage data and website entries by collecting de-identified data from the website during the one-month study.

Empowerment through the C.A.R.E. model was used to help participants remain engaged in the study. Each church’s ambassador was labeled as a “super-user”—a study participant who volunteered to help participants sync their wristband data and communicated hub issues to the research team. Super-users were usually able to give quick solutions and able to relay questions they could not answer with one phone call or email to the OC. Participants also provided buddy support to each other when syncing their data to the hubs, accessing their website, and encouraging each other to remain involved in the study. 

Participants received an incentive, a $25 gift card, for their completion of the data collection event and another $25 gift card if thirty days of PA data were received by the research team. At the completion of the study, participants were given the wristband to keep and provided with instructions on how to access their data using a mobile phone application (app) without them having to sync at a church. This allowed participants to continue to see their PA data when the hub was no longer available at the church.

## 3. Results

**Figure 2:**
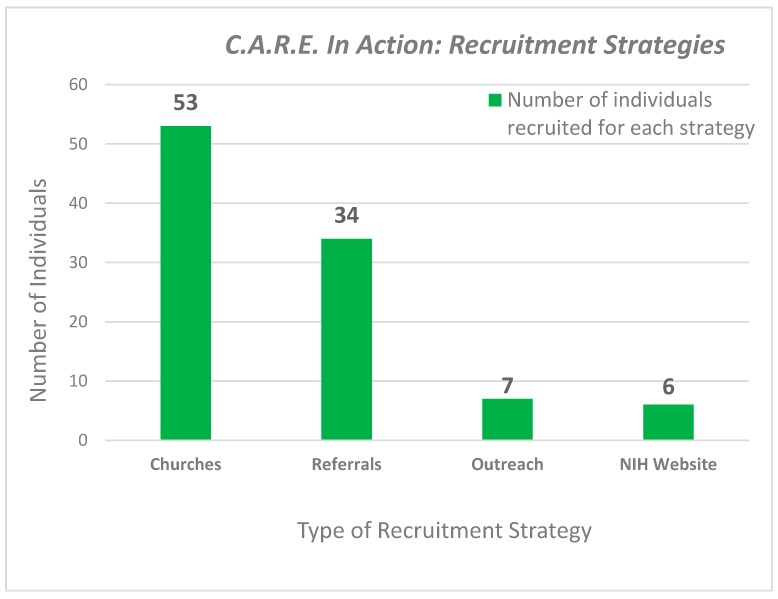
C.A.R.E in Action. Four types of recruitment strategies were used during this pilot.

Study participants were recruited by one or more recruitment strategies, (1) direct contact with the OC at churches and outreach activities, (2) referrals from community partners, (3) NIH patient recruitment website and (4) word-of-mouth (Table 1). Participants recruited directly by the OC were from contacts at community events, such as health fairs, block parties, and community meetings, where recruitment posters were on display, fliers were distributed, and presentations were made. Additionally, participants were recruited following church services; announcements were made during the church service and were printed in the church bulletin. Additional referrals to the study were made without any effort from the research team: NIH-recruitment website, community partners, participants, and word-of-mouth within the community. Our most successful recruitment, over 50%, came from our church partnerships, followed by participants referring their friends and family (Figure 2).

Over a thirteen-month period, between December 2013 and January 2014, ten outreach activities were performed and successfully distributed cardiovascular awareness information to over 1000 individuals in the community. One hundred twenty-five (125) people received personal cardiovascular risk using the Zuum Health Tracker App and 159 people were recruited for the Washington, D.C. Cardiovascular Health and Needs Assessment. Of the recruited participants, 100 enrolled in data collection activities with 99 individuals completing all required components of the health assessment data collection events. The study sample was 79% female and 99% African American. There were 58 recruited participants that were no-shows and one person arrived too late to participate. During the 30-day study period, 81 percent of the participants uploaded PA wristband data to a centralized hub at least once. 

The C.A.R.E. model was used throughout the study as we implemented our plan to keep enrolled participants engaged with the mHealth technology (Table 2). To encourage frequent communication, the OC engaged in weekly check-in phone conversations with the church ambassadors to identify any problems with the hubs located at the churches and/or reports of wearable device malfunction. Due to frequent contact, one malfunctioning device was quickly replaced, allowing for only a limited amount of data loss. It was necessary for us to support the WiFi at another church with a “hot spot” when the ambassador reported its slow response. 

## 4. Discussion

The C.A.R.E. model was successfully used as a tool to recruit and retain members of a high risk, limited resource and predominately African American community in Washington, D.C. The C.A.R.E. model was designed and implemented to strategically eliminate barriers for recruiting participants in this research study and was grounded in the principles of CBPR [15]. Implementing these strategies was influential in building a trusting relationship between the research team, the community, and participants. Intentional communication with study participants, the church leaders and their respective congregations was identified as a major factor in developing and maintaining excellent relationships. Recruiting more than our desired number of participants proved to be helpful in achieving our goal of 100 participants at the assessment events. It remained unknown to us as to why one participant left the event before completing all six stations; there were several unsuccessful attempts to communicate with this participant. 

### 4.1. The C.A.R.E. Model Addresses Difficulty with Recruitment and Retention

The literature is robust with discussions of known barriers to research participation for African American populations, but there is a scarcity of documented effective evidence-based strategies and practices regarding recruitment of minorities. The Tuskegee Syphilis Study has been identified as an important reason why many African Americans continue to distrust medical institutions and clinical research [16]. Furthermore, recent investigation has shown that African Americans tend to mistrust researchers and harbor beliefs that research may be published to negatively affect their community [17]. Participant recruitment and retention throughout the study could have been more difficult without the use of the C.A.R.E. model because it intentionally addresses one of the most consistent barriers to research participation for African American populations: distrust [17]. 

It can require additional investments of time and resources to learn which methods may work in African American communities to improve community acceptance of clinical research and thus improve participation. Prior studies suggest that researchers can be considered outsiders due to their cultural, educational, and economic backgrounds differing from the backgrounds of the group they are recruiting [18]. Our diverse research team was composed of individuals with similar backgrounds and/or understanding of urban African Americans to ensure that potential participants would relate and not view the researchers as outsiders. To overcome this barrier for the improvement of health outcomes, it is imperative that researchers utilize practices that consider the social and cultural aspects of the population they intend to study. Although there are governing principles and guidelines influenced by CBPR, there is no recommended model for community-based participatory researchers to follow when engaging African American research participants, so the C.A.R.E. model has the potential to fill that gap [15]. Although it was developed specifically for this targeted population, its strategies are not limited for use in African American populations.

The C.A.R.E. model provided a systematic approach to meet the community and gain their respect and willingness to participate in the study. We began our involvement in the targeted community by networking in churches, academic institutions, governments and health advocacy organizations where we recruited individuals interested in our research focus, obesity, to join the CAB. From these relationships, churches and participants were both recruited. Churches were an important component of this initiative as they have long been identified as an effective means of engaging with African American populations [19]. 

### 4.2. Implementation of C.A.R.E. Model May Be Especially Helpful When Launching Use of mHealth Technology

As mHealth technology becomes a more prevalent option for health equity, it is especially important that health researchers investigate its efficiency and efficacy in various populations with regards to geography, socioeconomic status, race/ethnicity, and culture. Recent research highlights that patients of lower socioeconomic status utilize mobile phones to a great extent and are interested in using mHealth technology to improve overall health [20,21]. The C.A.R.E. model provides a potential way to navigate the common hurdles that may come when attempting to engage a historically marginalized population with mHealth technologies. Implementation of the C.A.R.E. model may be especially helpful when launching use of mHealth technology in at-risk communities because many people in these communities may have misconceptions about the data collection and its usage and be apprehensive to utilize devices, which track information about their activities [21]. When investigating barriers to digital health engagement and recruitment, one group of researchers found that personal recommendations from trusted people in social groups was influential in potential participants engaging with technology [22,23]. The awareness component of the C.A.R.E. model ensured that participants were thoroughly educated on the data being collected and understood how it would be utilized. In addition, through the C.A.R.E. model and its relationship building, church members that were comfortable using technology were recruited to serve as Church Ambassadors to provide technologically inexperienced participants with technical support and guidance throughout the study; the availability and involvement of these church ambassadors as “super-users” empowered participants to remain engaged when they encountered difficulty or potentially had skepticism causing distrust.

### 4.3. Consideration of C.A.R.E. Model Employment in Future CBPR-Based Interventions

Community based participatory research (CBPR) principles have been shown to be a reliable reference framework for engaging diverse populations in research, but no documented work has been done to develop a specific model for use when implementing a CBPR approach [10,15]. Current literature has led to the development of a helpful CBPR conceptual model to guide research, but it does not offer practical action items for implementation [24]. Many researchers have found success by incorporating sites commonly frequented by the target population into their CBPR-based protocols and these sites are often faith-based organizations, barber shops and salons [13,25]. In addition, limited research has shown that utilizing CBPR principles may be effective for engaging diverse populations with mHealth technology. Cross-case analysis has shown that CBPR principles enhance research incorporating health information technology by allowing for more effective recruitment and retention of diverse populations and facilitating a wider research impact [26]. 

### 4.4. Limitations

One limitation of this study is the population size since the goal was only 100 participants. In addition, this study was limited by geographic location, but it is possible that this information may be applicable to other urban environments and produce similar results. In addition, since this study focused on a predominantly African American population, it has not been compared across diverse populations. Finally, the C.A.R.E. model has not been compared to other recruitment strategies, as this opportunity remains unavailable.

## 5. Conclusions

Thus, our findings suggest that the C.A.R.E. research recruitment model may be effective because the simple and practical framework based on guiding principles from CBPR and accounting for historical and cultural factors encountered by historically marginalized communities aided in building trust with our research participants. The suggested steps outlined in the C.A.R.E model respect the community, participants and the research team through ongoing communication and result in a long-term and potentially mutually beneficial relationship. By maintaining consistent and transparent communication between the research team and the community, we shared knowledge about cardiovascular disease, learned about community concerns and encouraged collaborative research. The C.A.R.E. model may be an effective tool which appropriately addresses barriers to recruiting, engaging and retaining African American research participants, and may be especially useful for various study types, especially those incorporating mHealth technology. Future research efforts should consider which types of outreach strategies are most cost effective, culturally sensitive and successful for recruitment. 

## Figures and Tables

**Table 1:** C.A.R.E. Recruitment Strategies. C.A.R.E. recruitment strategies included capitalizing on church partnerships, encouraging referrals and outreach, and posting on the NIH website.

Communication, Awareness, Relationship, Empowerment (C.A.R.E.) in Action: Recruitment Strategies		
Communication	Outreach	Participants recruited at community events such as: health fairs, block parties, health advocacy community meetings where recruitment posters were on display, fliers, distributed and presentations made.
Awareness	Referrals (word of mouth and community partners)	Participants referred to the study by: their friends and family who were participants, members of the community who learned about the study but were not enrolled themselves, community health advocacy groups.
Relationships	Churches	Participants recruited via: engagement following church services, announcements during services and printed in the church bulletin, handouts distributed.
Empowerment	NIH Website	Participants searched independently and discovered the study from the Healthy volunteer opportunity posted on the NIH Website.

Note: C.A.R.E.: Communication, Awareness, Relationships and Empowerment; NIH: National Institutes of Health. Each element of C.A.R.E. Research Recruitment model is color-coded to correspond with the model figure.

**Table 2:** C.A.R.E. Model as a useful tool for mHealth technology. The C.A.R.E strategies were important for introducing and maintaining engagement with mobile health technologies.

Participant Involvement and Engagement	Communication	Awareness	Relationships	Empowerment
Initial Involvement	Video-based tutorials Hands-on practice	Manuals for using devices Focus group to refine trial device and training tools	Stakeholders aware of devices and provide feedback Made clear about their role and purpose in the study	Appointed person for troubleshooting Hub in easily accessed location to facilitate physical activity tracking at church
Maintaining Engagement	Survey to assess barriers with use of device/facilitators to assist with use of the device Access to videos throughout the study	Follow up at one week to help sync at site Standardized method for troubleshooting device issues	Weekly follow-up calls and emails Gave device data/progress report at the first event of study	Learned how to use apps associated with device Ability to track physical activity even after the study

Note: C.A.R.E.: Communication, Awareness, Relationships and Empowerment. Each element of C.A.R.E. Research Recruitment model is color-coded to correspond with the model figure.

## Supplementary Materials

The following are available online at www.mdpi.com/1660-4601/14/11/1422/s1, Figure S1: Culturally tailored flyer used for community awareness and recruitment, Figure S2: Screenshot of the Zuum application used to provide cardiovascular health risk assessments.
