# Supplementary material for: The Communication, Awareness, Relationships and Empowerment (C.A.R.E.) Model: An Effective Tool for Engaging Urban Communities in Community-Based Participatory Research
Source: Int J Environ Res Public Health. 2017 Nov 21;14(11):1422. doi: 10.3390/ijerph14111422 (PMC5708061; doi:10.3390/ijerph14111422)
Supplement: Supplementary file 1 [file ijerph-14-01422-s001.pdf]

Supplementary Materials:

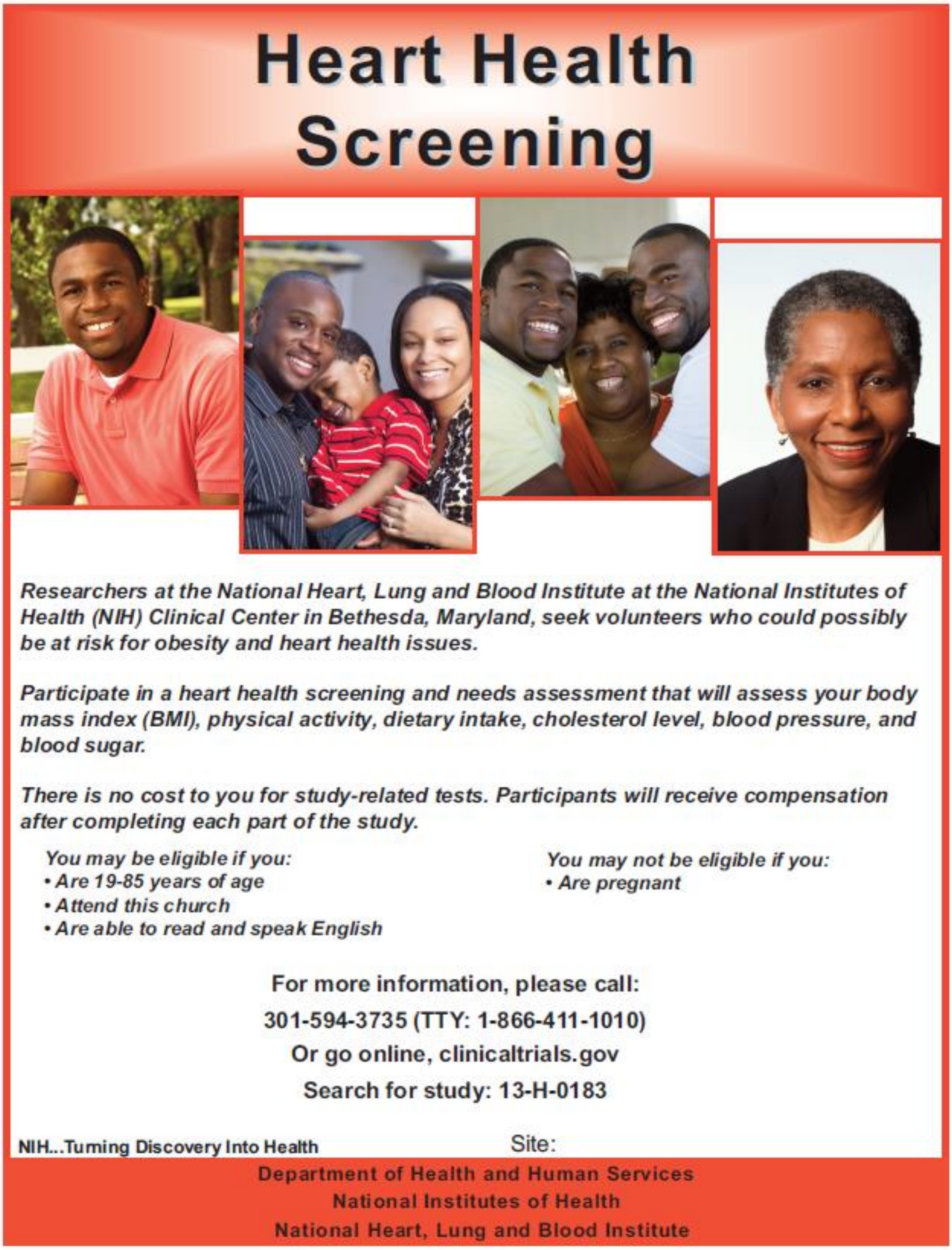

Figure 1. Culturally tailored flyer used for community awareness and recruitment.

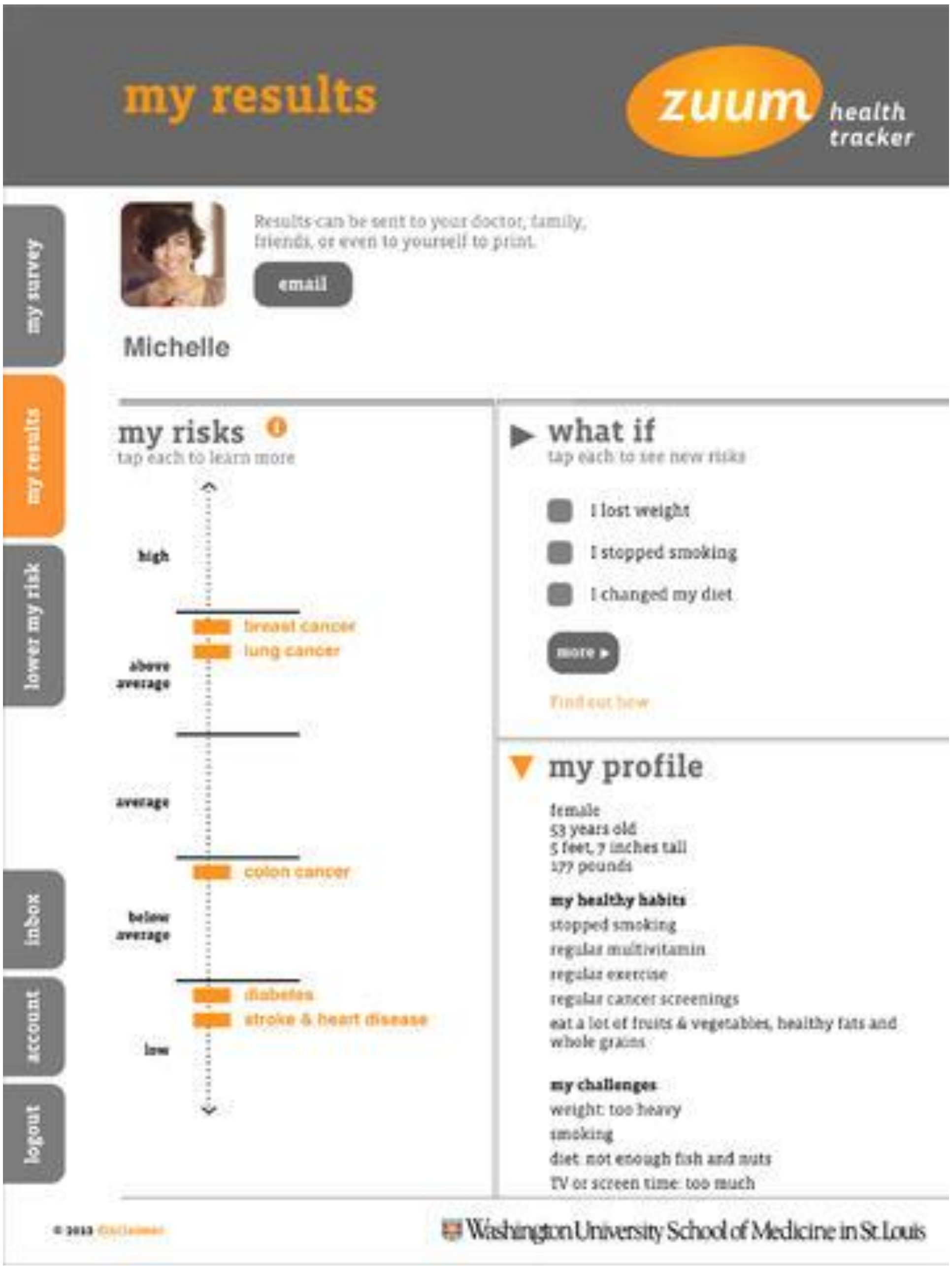

Figure 2. Screenshot of the Zuum application used to provide cardiovascular health risk assessments. (Please provide us a sufficiently high-resolution figures (minimum 1000 pixels width/height, or a resolution of 300 dpi or higher), we could not see the letters clearly.)
